# Supplementary material for: Fast Evaluation of Viral Emerging Risks (FEVER): A computational tool for biosurveillance, diagnostics, and mutation typing of emerging viral pathogens
Source: PLOS Glob Public Health. 2022 Feb 24;2(2):e0000207. doi: 10.1371/journal.pgph.0000207 (PMC10021650; doi:10.1371/journal.pgph.0000207)
Supplement: S1 Fig — Near-complete SARS-CoV-2 sequences (n = 299,404) were assessed from GISAID.org. The amplified regions of the FEVER assays (a) FEVER_5’UTR, (b) FEVER_Env, (c) FEVER_ORF1ab, and (d) FEVER_Spike and the U.S. CDC assays (e) N1 and (f) N2 were analyzed for SARS-CoV-2 variants. In each plot, no variants (N/A) were indicated by white and genomic positions containing variants were indicated by colored dots (green, blue, yellow or red). Plots were made using the Variant Visualizer that is under development and will be available at: https://cov.lanl.gov. (DOCX) [file pgph.0000207.s001.docx]

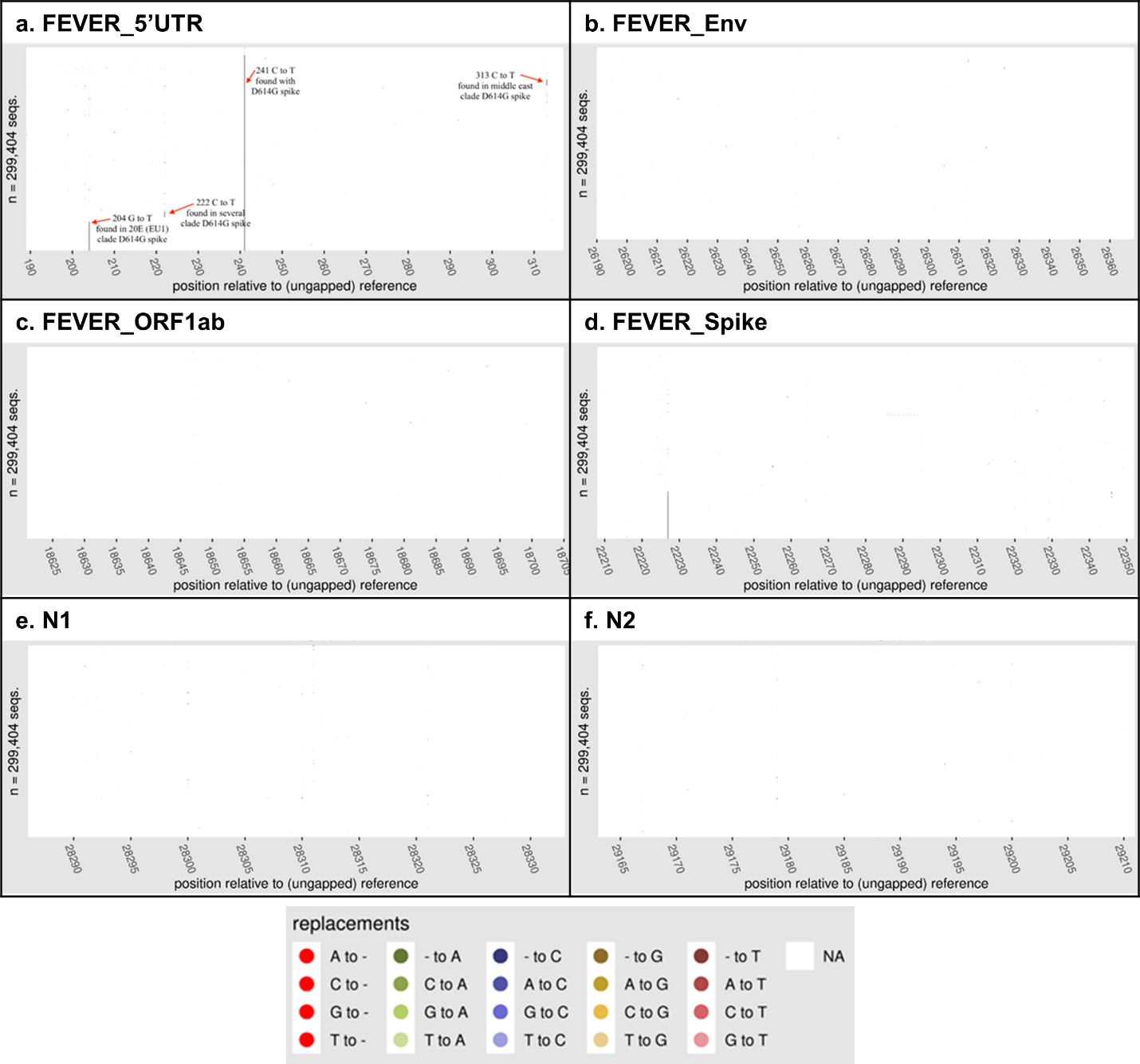


**S1 Fig. Visualization of SARS-CoV-2 variants in the amplified regions of FEVER and U.** **S. CDC RT-PCR assays.** Near-complete SARS-CoV-2 sequences (n = 299,404) were assessed from GISAID.org. The amplified regions of the FEVER assays (a) FEVER_5’UTR, (b) FEVER_Env, (c) FEVER_ORF1ab, and (d) FEVER_Spike and the U.S. CDC assays (e) N1 and (f) N2 were analyzed for SARS-CoV-2 variants. In each plot, no variants (N/A) were indicated by white and genomic positions containing variants were indicated by colored dots (green, blue, yellow or red). Plots were made using the Variant Visualizer that is under development and will be available at: <https://cov.lanl.gov>
