# Supplementary material for: Fast Evaluation of Viral Emerging Risks (FEVER): A computational tool for biosurveillance, diagnostics, and mutation typing of emerging viral pathogens
Source: PLOS Glob Public Health. 2022 Feb 24;2(2):e0000207. doi: 10.1371/journal.pgph.0000207 (PMC10021650; doi:10.1371/journal.pgph.0000207)
Supplement: S1 Table — (DOCX) [file pgph.0000207.s002.docx]

**S1 Table. Patient demographics.**

| **Variables** | **No.** |
| --- | --- |
| *Age range (years)* | |
| <20 | 1 |
| 20-40 | 13 |
| 40-60 | 42 |
| >60 | 44 |
| *Race/Ethnicity* | |
| Asian/Pacific Islander | 3 |
| Black/African American | 1 |
| Hispanic/Latino | 35 |
| Missing/Not reported | 6 |
| Native American | 24 |
| Other | 1 |
| White | 30 |
