# Supplementary material for: Fast Evaluation of Viral Emerging Risks (FEVER): A computational tool for biosurveillance, diagnostics, and mutation typing of emerging viral pathogens
Source: PLOS Glob Public Health. 2022 Feb 24;2(2):e0000207. doi: 10.1371/journal.pgph.0000207 (PMC10021650; doi:10.1371/journal.pgph.0000207)
Supplement: S3 Table — (DOCX) [file pgph.0000207.s004.docx]

**S3 Table. Characterization of the D614G mutation among 59 SARS-CoV-2 positive nasopharyngeal swab samples.**

| **Sample #** | **FEVER_ORF1ab** | **FEVER_Spike** | **G614 Cт** | **D614 Cт** | **SNP call** |
| --- | --- | --- | --- | --- | --- |
| 1 | Positive | Positive | 33.54 | 36.09 | G614 |
| 2 | Positive | Positive | 24.55 | 26.92 | G614 |
| 3 | Positive | Positive | 12.95 | 15.27 | G614 |
| 6 | Positive | Positive | 25.93 | 28.67 | G614 |
| 7 | Positive | Positive | 31.81 | 34.16 | G614 |
| 8 | Positive | Positive | 31.99 | 34.62 | G614 |
| 9 | Positive | Positive | 18.69 | 21.64 | G614 |
| 15 | Positive | Positive | 23.77 | 26.64 | G614 |
| 16 | Positive | Positive | 22.41 | 24.93 | G614 |
| 17 | Positive | Positive | 24.74 | 27.51 | G614 |
| 18 | Not detected | Positive | 38.68 | 41.46 | G614 |
| 19 | Positive | Positive | 23.21 | 25.71 | G614 |
| 20 | Positive | Positive | 25.97 | 28.83 | G614 |
| 21 | Positive | Positive | 30.69 | 33.27 | G614 |
| 22 | Not detected | Positive | 27.56 | 30.31 | G614 |
| 23 | Positive | Not detected | 25.20 | 27.71 | G614 |
| 24 | Positive | Positive | 34.50 | 36.95 | G614 |
| 25 | Positive | Not detected | 25.91 | 28.58 | G614 |
| 28 | Positive | Not detected | 21.64 | 24.38 | G614 |
| 29 | Positive | Not detected | 29.69 | 32.41 | G614 |
| 30 | Positive | Positive | 37.62 | 40.72 | G614 |
| 31 | Positive | Positive | 30.84 | 33.54 | G614 |
| 33 | Positive | Positive | 14.38 | 17.16 | G614 |
| 35 | Positive | Positive | 34.30 | 36.78 | G614 |
| 36 | Positive | Positive | 19.00 | 21.86 | G614 |
| 37 | Positive | Positive | 27.32 | 29.89 | G614 |
| 38 | Positive | Positive | 15.96 | 18.64 | G614 |
| 39 | Positive | Positive | 17.52 | 20.23 | G614 |
| 40 | Positive | Positive | 32.84 | 35.60 | G614 |
| 42 | Positive | Positive | 29.46 | 32.00 | G614 |
| 43 | Positive | Positive | 34.50 | 37.24 | G614 |
| 44 | Positive | Positive | 29.90 | 32.76 | G614 |
| 46 | Positive | Not detected | 31.98 | 34.90 | G614 |
| 49 | Positive | Not detected | 35.34 | 38.11 | G614 |
| 50 | Positive | Positive | 33.47 | 36.36 | G614 |
| 52 | Positive | Positive | 35.60 | 38.36 | G614 |
| 54 | Positive | Positive | 33.62 | 36.43 | G614 |
| 55 | Positive | Not detected | 39.91 | 44.01 | G614 |
| 57 | Positive | Not detected | 37.39 | 41.02 | G614 |
| 58 | Positive | Positive | 27.05 | 30.97 | G614 |
| 59 | Positive | Positive | 32.81 | 36.61 | G614 |
| 60 | Positive | Positive | 21.72 | 25.52 | G614 |
| 63 | Positive | Not detected | 34.80 | 38.69 | G614 |
| 64 | Positive | Not detected | Not detected | Not detected | Not detected |
| 66 | Not detected | Positive | 29.46 | 33.33 | G614 |
| 67 | Positive | Positive | 19.23 | 23.00 | G614 |
| 73 | Positive | Positive | 33.84 | 36.52 | G614 |
| 76 | Positive | Positive | 19.34 | 21.92 | G614 |
| 77 | Positive | Positive | 37.29 | 40.39 | G614 |
| 78 | Positive | Positive | 24.74 | 27.25 | G614 |
| 80 | Positive | Positive | 25.70 | 28.39 | G614 |
| 83 | Positive | Positive | 35.48 | 38.16 | G614 |
| 88 | Positive | Positive | 32.01 | 34.85 | G614 |
| 89 | Positive | Positive | 25.03 | 27.70 | G614 |
| 91 | Positive | Positive | 28.52 | 31.25 | G614 |
| 92 | Positive | Positive | 15.59 | 18.35 | G614 |
| 95 | Positive | Positive | 33.65 | 36.51 | G614 |
| 97 | Positive | Positive | Not detected | Not detected | Not detected |
| 99 | Positive | Positive | Not detected | Not detected | Not detected |

Abbreviations: C_T_, cycle threshold; SNP, single-nucleotide polymorphism.
