# Supplementary material for: Fast Evaluation of Viral Emerging Risks (FEVER): A computational tool for biosurveillance, diagnostics, and mutation typing of emerging viral pathogens
Source: PLOS Glob Public Health. 2022 Feb 24;2(2):e0000207. doi: 10.1371/journal.pgph.0000207 (PMC10021650; doi:10.1371/journal.pgph.0000207)
Supplement: S4 Table — (DOCX) [file pgph.0000207.s005.docx]

**S4 Table. Sequence confirmation of the D614G mutation among 17 nasopharyngeal swab samples.**

| **Sample** | **Sequence (23,353-23,452)** |
| --- | --- |
| MN985325  (reference) | TATAACACCAGGAACAAATACTTCTAACCAGGTTGCTGTTCTTTATCAGG**A**TGTTAACTGCACAGAAGTCCCTGTTGCTATTCATGCAGATCAACTTACT |
| 1 | TATAACACCAGGAACAAATACTTCTAACCAGGTTGCTGTTCTTTATCAGG**G**TGTTAACTGCACAGAAGTCCCTGTTGCTATTCATGCAGATCAACTTACT |
| 2 | TATAACACCAGGAACAAATACTTCTAACCAGGTTGCTGTTCTTTATCAGG**G**TGTTAACTGCACAGAAGTCCCTGTTGCTATTCATGCAGATCAACTTACT |
| 3 | TATAACACCAGGAACAAATACTTCTAACCAGGTTGCTGTTCTTTATCAGG**G**TGTTAACTGCACAGAAGTCCCTGTTGCTATTCATGCAGATCAACTTACT |
| 6 | TATAACACCAGGAACAAATACTTCTAACCAGGTTGCTGTTCTTTATCAGG**G**TGTTAACTGCACAGAAGTCCCTGTTGCTATTCATGCAGATCAACTTACT |
| 8 | TATAACACCAGGAACAAATACTTCTAACCAGGTTGCTGTTCTTTATCAGG**G**TGTTAACTGCACAGAAGTCCCTGTTGCTATTCATGCAGATCAACTTACT |
| 9 | TATAACACCAGGAACAAATACTTCTAACCAGGTTGCTGTTCTTTATCAGG**G**TGTTAACTGCACAGAAGTCCCTGTTGCTATTCATGCAGATCAACTTACT |
| 16 | TATAACACCAGGAACAAATACTTCTAACCAGGTTGCTGTTCTTTATCAGG**G**TGTTAACTGCACAGAAGTCCCTGTTGCTATTCATGCAGATCAACTTACT |
| 17 | TATAACACCAGGAACAAATACTTCTAACCAGGTTGCTGTTCTTTATCAGG**G**TGTTAACTGCACAGAAGTCCCTGTTGCTATTCATGCAGATCAACTTACT |
| 18 | TATAACACCAGGAACAAATACTTCTAACCAGGTTGCTGTTCTTTATCAGG**G**TGTTAACTGCACAGAAGTCCCTGTTGCTATTCATGCAGATCAACTTACT |
| 21 | TATAACACCAGGAACAAATACTTCTAACCAGGTTGCTGTTCTTTATCAGG**G**TGTTAACTGCACAGAAGTCCCTGTTGCTATTCATGCAGATCAACTTACT |
| 22 | TATAACACCAGGAACAAATACTTCTAATCAGGTTGCTGTTCTTTATCAGG**G**TGTTAACTGCACAGAAGTCCCTGTTGCTATTCATGCAGATCAACTTACT |
| 30 | TATAACACCAGGAACAAATACTTCTAACCAGGTTGCTGTTCTTTATCAGG**G**TGTTAACTGCACAGAAGTCCCTGTTGCTATTCATGCAGATCAACTTACT |
| 52 | TATAACACCAGGAACAAATACTTCTAACCAGGTTGCTGTTCTTTATCAGG**G**TGTTAACTGCACAGAAGTCCCTGTTGCTATTCATGCAGATCAACTTACT |
| 59 | TATAACACCAGGAACAAATACTTCTAACCAGGTTGCTGTTCTTTATCAGG**G**TGTTAACTGCACAGAAGTCCCTGTTGCTATTCATGCAGATCAACTTACT |
| 73 | TATAACACCAGGAACAAATACTTCTAACCAGGTTGCTGTTCTTTATCAGG**G**TGTTAACTGCACAGAAGTCCCTGTTGCTATTCATGCAGATCAACTTACT |
| 78 | TATAACACCAGGAACAAATACTTCTAACCAGGTTGCTGTTCTTTATCAGG**G**TGTTAACTGCACAGAAGTCCCTGTTGCTATTCATGCAGATCAACTTACT |
| 92 | TATAACACCAGGAACAAATACTTCTAACCAGGTTGCTGTTCTTTATCAGG**G**TGTTAACTGCACAGAAGTCCCTGTTGCTATTCATGCAGATCAACTTACT |

The spike gene from nucleotide position 23,353 to 23,452 (relative to reference sequence USA-WA1/2020 accession number MN985325) is displayed with A23403G (D614G amino acid change) mutation in red.
